# Can ultrasound contrast agents increase the treatment envelope?

**DOI:** 10.1186/2050-5736-3-S1-O30

**Published:** 2015-06-30

**Authors:** Nathan McDannold, Natalia Vykhodtseva, Costas Arvanitis, Margaret Livingstone, Ferenc Jolesz

**Affiliations:** 1 Brigham & Women’s Hospital, Boston, Massachusetts, United States; 2 Harvard Medical School, Boston, MA, United States

## Background/introduction

Currently, thermal ablation via transcranial MRI-guided focused ultrasound is restricted to centrally-located regions in the brain. This limitation is the result of skull heating when the focal region is steered to more peripheral regions in the brain, along with other factors such as limitations in beam steering in the currently-available clinical brain focused ultrasound systems. The treatment envelope could be expanded if the time averaged acoustic power needed for ablation could be reduced.

## Methods

The introduction of ultrasound contrast agents – preformed microbubbles that are injected intravenously – can reduce the power needed to ablate tissue. These commercially-available microbubble agents respond strongly to an acoustic field, even at low intensities, and greatly magnify the resulting bioeffects.

## Results and conclusions

This presentation will provide a summary of animal data obtained in small animals and in nonhuman primates at our institution and by others that tested contrast-enhanced ultrasound ablation. Overall, these studies have demonstrated that ablation can be achieved at time-averaged acoustic power levels at least an order of magnitude less than what is needed for thermal ablation.

The huge reduction in acoustic power that can be achieved with ablation combined with ultrasound contrast agents may enable a substantial increase in the “treatment envelope” for transcranial focused ultrasound systems. New methods are being developed to control the procedure and to ensure that unwanted tissue effects do not occur outside of the focal region. These developments, along with other unresolved issues with this mode of ablation will be topics for discussion.

